# Calcium supplementation during pregnancy and long‐term offspring outcome: a systematic literature review and meta‐analysis

**DOI:** 10.1111/nyas.14729

**Published:** 2022-01-03

**Authors:** Päivi Korhonen, Kati Tihtonen, Jaana Isojärvi, Riitta Ojala, Ulla Ashorn, Per Ashorn, Outi Tammela

**Affiliations:** ^1^ Department of Pediatrics Tampere University Hospital Tampere Finland; ^2^ Faculty of Medicine and Health Technology, Center for Child, Adolescent, and Maternal Health Research Tampere University Tampere Finland; ^3^ Department of Obstetrics and Gynecology Tampere University Hospital Tampere Finland; ^4^ Library Tampere University Tampere Finland

**Keywords:** calcium supplementation, child, pregnancy, offspring, blood pressure, growth

## Abstract

The World Health Organization currently recommends calcium supplementation for pregnant women, especially those with low calcium intakes, to reduce the risk of hypertension and preeclampsia. We aimed to evaluate the effect of this intervention on selected offspring outcomes. A systematic search was conducted in 11 databases for published randomized controlled trials (RCTs) on the effect of maternal calcium supplementation with or without vitamin D during pregnancy on selected offspring cardiovascular, growth, and metabolic and neurodevelopmental outcomes. Screening of titles and abstracts of 3555 records and full texts of 31 records yielded six RCTs (nine reports, *n* = 1616). Forest plot analyses were performed if at least two studies presented comparable data on the same outcome. In one study (*n* = 591), high‐dose calcium supplementation during pregnancy was associated with a decreased risk of offspring high systolic blood pressure at 5–7 years of age (risk ratio = 0.59; 95% confidence interval: 0.39–0.90). The effects of the intervention on offspring growth, metabolic, and neurodevelopmental outcomes remain unknown because of conflicting or insufficient data. High risk of attrition bias decreased the quality of the evidence. Limited available data from RCTs do not provide sufficient evidence to conclude that prenatal calcium supplementation influences offspring health outcomes beyond the newborn period.

## Graphical abstract

The WHO currently recommends calcium supplementation for pregnant women, especially those with low calcium intakes, to reduce the risk of hypertension and preeclampsia. We aimed to evaluate the effect of this intervention on selected offspring outcomes. A systematic search was conducted in 11 databases for published randomized controlled trials on the effect of maternal calcium supplementation with or without vitamin D during pregnancy on selected offspring cardiovascular, growth, metabolic, and neurodevelopmental outcomes.

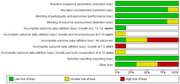

## Introduction

Insufficient calcium intake during pregnancy has been linked to the development of maternal hypertension, an important cause of maternal morbidity, fetal growth restriction (FGR), and preterm birth.^1^ Preeclampsia is a pregnancy complication characterized by high blood pressure (BP) and signs of damage to another organ system, most often the kidneys and the liver. The World Health Organization (WHO) has recommended calcium supplementation of 1500–2000 mg/day for the prevention of preeclampsia from 20 weeks of gestation to all pregnant women in areas of low dietary calcium intake.^2^ A recently updated Cochrane review supported this recommendation.^3^ The same review suggested a preventive effect of calcium supplementation against preterm birth but found few data on long‐term offspring outcome.^3^

Vitamin D is essential for calcium and bone metabolism, and a poor vitamin D status during pregnancy may impair calcium utilization in the body. Studies using the combination of calcium and vitamin D supplementation have been included also in the previous systematic reviews on the effects of maternal calcium supplementation during pregnancy.^3^ The WHO does not currently recommend routine vitamin D supplementation for all pregnant women because of the lack of data on maternal vitamin D levels that affect bone mass in the offspring, the optimal initiation of supplementation, and the duration of its effects.^4^

Maternal preeclampsia has been suggested to result in adverse health consequences also to offspring,^5^ such as higher BP in childhood or adolescence,^6^, ^7^, ^8^, ^9^ increased risk of cerebral palsy,^10^ and increased risk of stroke in adulthood.^11^ The roles of preeclampsia, preterm birth, and FGR as risk factors of future health are intertwined. Earlier work suggests that FGR, often related to maternal preeclampsia, is a major risk factor for stunting in infancy and may predispose affected children to cardiovascular disease and metabolic syndrome in adulthood.^12^ However, others have observed that neither FGR nor childhood growth trajectories could explain the association between preeclampsia and high childhood BP in the offspring.^13^ Instead, the effects of the nutritional environment during fetal life on offspring outcome might be associated with changes in the epigenetic programming of growth, body composition, and cardiovascular and metabolic health.^14^, ^15^

Interventions associated with a decreased risk of preeclampsia and preterm birth might also be associated with a decreased risk of adverse health consequences of these conditions to the offspring. The aim of our review was to synthesize existing evidence from randomized trials on the impact of maternal calcium supplementation during pregnancy on long‐term cardiovascular, growth, metabolic, and neurodevelopmental outcomes of the offspring. The review will hopefully help to improve and update global and national guidelines as well as identify priorities for future research on the subject.

## Materials and methods

The study protocol was planned according to the Preferred Reporting Items for Systematic Review and Meta‐Analysis Protocols (PRISMA‐P)^16^ and registered in PROSPERO July 5th, 2020 (CRD 42020176152).

We searched for randomized controlled trials (RCTs), including cluster‐randomized studies on pregnant women and their offspring. The primary target settings were South Asia and Sub‐Saharan Africa, but given the lack of data, the search was expanded to all regions. Interventions included supplementary calcium alone or with vitamin D during pregnancy, started at 35 gestational weeks at the latest and stopped at delivery. We accepted studies using elemental calcium doses up to the maximum of 2000 mg/day^17^ and vitamin D (vitamin D, cholecalciferol, or ergocholecalciferol) doses up to the maximum of 4000 IU (100 μg)/day.^18^ Studies on food fortification were excluded. We included studies with control groups using placebo, no treatment, standard care, or regular diet.

We accepted studies evaluating offspring cardiovascular, growth, metabolic, and neurodevelopmental outcomes from 2 weeks to 18 years of age. Primary outcomes comprised BP, height, weight, glucose intolerance, plasma lipid profile, cerebral palsy, developmental delay, intellectual impairment, and behavioral/learning difficulties. Secondary outcomes included mid‐upper arm circumference (MUAC), skinfold thickness, lean body mass, overweight, and head circumference.

### Search strategy

An experienced information specialist (J.I.) conducted the search on September 7, 2020 in MEDLINE (via OvidSP), Cochrane Central Register of Controlled Trials (CENTRAL, via Wiley Cochrane Library), CINAHL Complete (via EBSCOhost), Scopus, Science Citation Index (via Web of Science), Social Science Citation Index (via Web of Science), Conference Proceedings Citation Index ‐ Science (via Web of Science), Conference Proceedings Citation Index‐ Social Science & Humanities (via Web of Science), ClinicalTrials.gov, WHO International Clinical Trials Registry Platform (WHO ICTRP), and PROSPERO.

Strategies for identifying relevant reports of RCTs comprised both database‐specific subject headings (e.g., Medical Subject Headings (MeSHs)) and terms that are likely to appear in study titles and abstracts (Appendix S1, online only). We applied published search filters to inform the strategies in MEDLINE^19^ and CINAHL.^20^ We did not include animal studies. Publication types unlikely to provide relevant information, such as editorials and news, were excluded where possible. No other limits were applied. We loaded the results into EndNote (version X9.3, Clarivate Analytics) for deduplication.

### Study selection

Two review authors (K.T. and P.K.) independently screened the titles and abstracts of the records, as well as full texts of studies eligible for outcome analysis, using Covidence, online software for streamlining systematic review processes. Disagreements were resolved through discussion. Reasons for exclusion of the studies during full‐text screening were recorded. We screened the reference lists of relevant reviews for any additional applicable studies. No additional studies were identified. Neither of the review authors was blinded to the journal titles, study authors, or institutions. A PRISMA flow chart was generated.

Two review authors (K.T. and P.K.) independently extracted data using Covidence, resolving disagreements by discussion. The extracted data included (1) author, publication year, country, and area; (2) study design; (3) characteristics of the study population and controls (number of randomized and analyzed, age, parity, and baseline dietary intake for calcium); (4) description and duration of intervention(s); (5) outcome measurement(s); and (6) effect measure(s), if applicable.

### Risk of bias

Two review authors (K.T. and P.K.) evaluated independently the risk of bias using the Cochrane risk‐of‐bias tool for randomized trials in Covidence with the following domains: sequence generation, allocation concealment, blinding of participants and personnel, blinding of outcome assessors, incomplete outcome data (attrition bias), selective outcome reporting, and other bias. Each domain was graded as having low, unclear, or high risk of bias; disagreements were resolved by discussion. Studies with some concern as well as lack of information on the risk of bias were graded as having unclear risk of bias.^21^

For follow‐up studies of trials originally designed for evaluating maternal outcomes, two risk‐of‐bias domains (sequence generation and allocation concealment) were assessed from the original trials. Follow‐up reports on the same original study population were merged for the risk‐of‐bias evaluation. Despite this, risk‐of‐bias domains were assessed for different outcomes and follow‐up ages separately, when appropriate.

### Data analysis

We exported the data extracted using Covidence to the RevMan 5.4 program for further analyses. For dichotomous outcomes, we planned to determine the risk ratio (RR) with 95% confidence interval (CI). Continuous outcomes were analyzed using mean differences with 95% CI. Data presented separately for males and females were combined in RevMan using appropriate methods.

We performed forest plot analyses for outcome parameters with comparable data from at least two different studies, analyzing the data according to age groups. We used fixed‐effect meta‐analysis for combining information, where it was reasonable to assume that studies were estimating the same underlying treatment effect. The heterogeneity of the studies in the meta‐analysis was assessed using the I^2^, a Chi‐squared test, and Tau.^2^ In case of heterogeneity across the studies (I^2^ > 50%, Tau^2^ > 0, or *P* < 0.10), we used random‐effects meta‐analysis.

We planned to do subgroup analyses, if appropriate data were available, according to the dose of calcium (low dose: less than 1000 mg/day; high dose: 1000 mg–2000 mg/day), calcium supplementation alone and with vitamin D, women or populations with low dietary calcium intake (as defined by trial authors, or if not defined, mean daily intake less than 900 mg), geographical areas (Sub‐Saharan Africa and South Asia), teenagers, and multiparas.

### Interpretation of evidence

We interpreted the available evidence according to the coding published by Koivu and associates.^22^ The categories included positive effect, possible positive effect, no positive effect, and unknown effect due to insufficient or inconclusive published research (Appendix S2, online only).

## Results

### Study selection

**Figure 1:**
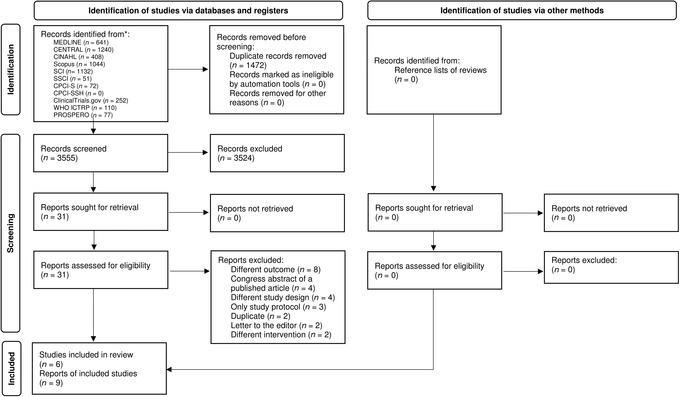
Selection of the randomized controlled trials for the review.

Literature searches retrieved 5027 records in total, and 3555 records remained for screening after deduplication. After title and abstract screening, 31 records were eligible for full text review. Finally, the search yielded six studies^23^, ^24^, ^25^, ^26^, ^27^, ^28^ (nine reports^23^, ^24^, ^25^, ^26^, ^27^, ^28^, ^29^, ^30^, ^31^) for data extraction (Fig. 1 and Table 1).

**Table 1:** Characteristics of the included randomized controlled studies

Original reference	Belizán *et al*.^32^	Levine *et al*.^33^	Crowther *et al*.^34^	Villar *et al*.^35^	Goldberg *et al*.^23^				Diogenes *et al*.^36^
Country, year	Argentina, years not stated	The United States (CPEP trial), years not stated	Australia (Australian Calcium Trial), 1992−1996	Egypt (subset study of the WHO trial), 2001−2003	The Gambia, 1995−2000				Brazil, 2009−2011
Number of originally randomized (Ca/placebo), setting	593/601, selected private hospital (309/305)	2295/2294, selected university center (497 in all)	227/229, five maternity hospitals	700 in all, 200 eligible for follow‐up, one maternity center	330/332, 16 rural villages				43/41, adolescents
Daily dose of calcium and vit. D	2000 mg Ca	2000 mg Ca	1800 mg Ca	1500 mg Ca	1500 mg Ca				600 mg Ca + 200 IU vit. D
Calcium started	20 weeks of gestation	13−21 weeks of gestation	20 weeks of gestation	20 weeks of gestation	20 weeks of gestation				26 weeks of gestation
Original/primary outcome	Hypertensive disorders of pregnancy	Hypertensive disorders of pregnancy	Hypertensive disorders of pregnancy and preterm delivery	Hypertensive disorders of pregnancy and preterm delivery	Hypertensive disorders of pregnancy and infant growth during the first year (secondary outcome)				Maternal bone mass and bone‐ and calcium‐related hormones during lactation
Offspring outcome reference	Belizán *et al*.^24^	Hatton *et al*.^25^	Hiller *et al*.^26^	Abdel‐Aleem *et al*.^27^	Goldberg *et al*.^23^	Hawkesworth *et al*.^29^	Ward *et al*.^30^	Prentice *et al*.^31^	Diogenes *et al*.^28^
*n* (Ca/placebo)	257/261	3 months: 130/130 2 years: 35/18	91/88	6 months: 17/25 9 months: 44/39 12 months: 17/17	257/263	179/171	223/224	141/149	30/26
Offspring age	5−9 years	3 months, 2 years	4−7 years	6, 9, and 12 months	2, 13, and 52 weeks	5−10 years	8−12 years	5−10 years	5 weeks
Offspring outcomes									
‐ BP	BP	BP	BP			BP			
‐ Height/weight	Height/weight			Length/weight	Length/weight	Height/weight	Height/weight	Height/weight,	Length/weight
‐ MUAC				MUAC	MUAC		MUAC	MUAC	
‐ Skinfold thickness					TST		TST	TST	
‐ Lean mass							Lean mass		
‐ HC				HC	HC				
‐ Other	BMI			Fetal growth		BMI, fat mass index, and trunk fat	HAZ, fat mass, and bone measurements	BMI, IGF1, IGFBP3, leptin, insulin, and Ca‐related indices	Bone measurements

BMI, body mass index; BP, blood pressure; Ca, calcium; HAZ, height‐associated Z‐score; HC, head circumference; MUAC, mid‐upper arm circumference; TST, triceps skinfold thickness.

### Study characteristics

The included trials comprised 809 participants in the calcium group and 807 in the placebo group (Table 1). The study populations were originally derived from six large randomized trials, where the primary objectives were to examine the effect of calcium supplementation during pregnancy on hypertensive disorders in pregnancy in three studies^23^, ^32^, ^33^ and on pregnancy hypertension and preterm delivery in two studies.^34^, ^35^ Maternal bone mass and bone metabolism during lactation was the primary maternal outcome in one study.^36^ In addition, one study assessed infant growth during the first year of life as a secondary outcome^23^ and provided the basis for three follow‐up studies^29^, ^30^, ^31^ included in our review (Table 1).

Four of the nine reports (including one follow‐up report of the Gambian study^23^) studied offspring BP as main outcome measures,^24^, ^25^, ^26^, ^29^ three studies assessed growth parameters,^27^, ^28^, ^30^ and offspring IGF1 was the primary outcome in one study^31^ (Table 1). No RCT reports were found on the impact of maternal calcium supplementation during pregnancy on glucose intolerance, lipid profile, overweight, cerebral palsy, developmental delay, intellectual impairment, or behavioral/learning difficulties in the offspring.

One trial^23^ and its three follow‐up studies^29^, ^30^, ^31^ were from the Gambia, and the other studies were from Argentina, the United States, Australia, Egypt, and Brazil (Table 1). No studies were from South Asia. In the Gambian, Brazilian, and Egyptian study areas, maternal calcium intakes were 300–400 mg/day^23^, ^29^, ^30^, ^31^ and less than 600 mg/day^27^, ^28^ at baseline. The Australian study^26^ reported intakes below 800 mg/day in 14.4–16% in the participating mothers. The Argentinean study^24^ reported no data on baseline maternal calcium intake.

All studies used calcium carbonate supplements, with dosages of 600,^28^ 1500,^23^, ^29^, ^30^, ^31^ 1800,^26^ and 2000 mg/day.^24^, ^25^ The doses were reported as elemental calcium in the studies from the Gambia^23^, ^29^, ^30^, ^31^ and the United States.^25^ In most studies, the intervention included high‐dose calcium alone, starting at 21 weeks of gestation at the latest and stopped at delivery. In one study, adolescent pregnant women received low‐dose calcium supplementation combined with vitamin D during the third trimester.^28^ Data on maternal compliance were available in six studies,^23^, ^24^, ^25^, ^26^, ^28^, ^29^ the compliances being approximately 65% in one study,^25^ 80–90% in three studies,^24^, ^26^, ^28^ and almost 100% in two^23^, ^29^ studies. One study^27^ did not report maternal compliance during follow‐up, but it was approximately 85% in the original study population.^35^ No extensive subgroup analyses could be performed because of insufficient data.

### Risk of bias

**Figure 2:**
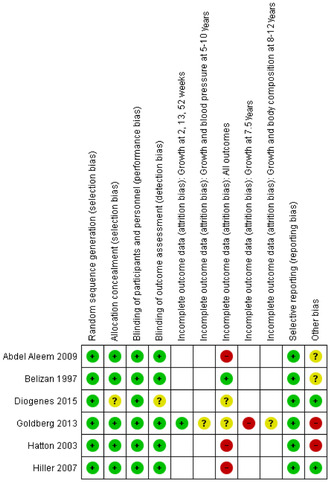
Risk‐of‐bias summary. Review authors' judgments about each risk‐of‐bias item for each included study. Risk‐of‐bias domains of Refs. 29, 30, 31 are reported under Goldberg *et al*.,^23^ because of merging of studies from the same original study population in Covidence. Empty cells indicate “not applicable.”

All studies were assessed as having a low risk of bias in four to six domains of the seven that were evaluated (Fig. 2). One study was graded as having a high risk of bias in two domains, and two studies in one domain. One study was graded as having unknown risk of bias in three domains, and two studies in one domain (Fig. 2).

**Figure 3:**
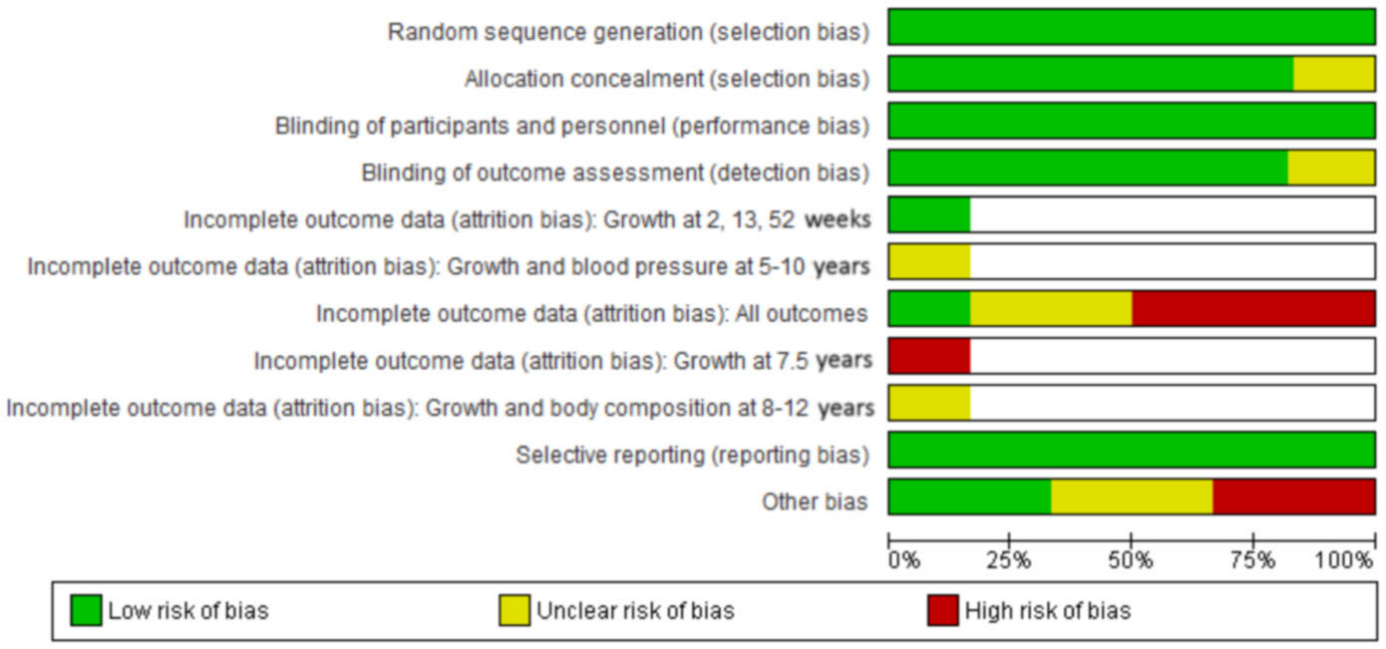
Risk‐of‐bias graph. Review authors' judgments about each risk of bias item presented as percentages across all included studies. Risk of attrition bias for Refs. 23 and 29–31, which originate from the same study population, is also presented by outcome and age of the child.

The general risk of attrition bias was graded as high in three studies, which reported missing data of 47–79%,^27^ 48–89%,^25^ and 57%,^26^ and as unknown in two studies (Fig. 3). In addition, a high risk of attrition bias (missing data = 56%) was observed in one Gambian follow‐up study.^31^ In one study, pregnant women were originally randomized in four hospitals^32^ but only one hospital was included in the follow‐up study.^24^ In this study, 87.6% of the eligible mothers and children participated, so the risk of attrition bias was low. The participating women were older and taller, and had higher mean BPs than the population in the original study. However, the authors considered the characteristics of the mothers whose children were eligible and of those whose children were assessed as being closely similar at randomization. In the Australian study,^26^ the participants were older, had higher calcium intakes, were more likely to be compliant with the trial intervention, and less likely to be smokers than the nonparticipants. In addition, infants of the participants in the calcium group were more likely to be small for gestational age than the offspring of nonparticipants.

Two studies were assessed as having high and two as having unknown risk of bias in the domain “other bias” (Figs. 2 and 3). This included lack of information on possible effect‐modifying factors (children's dietary calcium and vitamin D intake, and physical activity) concerning long‐term outcomes (Fig. 2).

### Effect of maternal calcium supplementation on offspring outcomes

#### Primary outcomes

##### Blood pressure

Data on offspring BP were available from four trials from Argentina,^24^ the United States,^25^ Australia,^26^ and the Gambia,^29^ including 657 children in the calcium and 650 in the placebo group (Tables 1 and 2).

**Table 2:** The effect of calcium supplementation during pregnancy on selected offspring outcomes in comparison with placebo

Reference	Belizán *et al*.^24^	Hatton *et al*.^25^	Hiller *et al*.^26^	Abdel‐Aleem *et al*.^27^	Goldberg *et al*.^23^	Hawkesworth *et al*.^29^	Ward *et al*.^30^	Prentice *et al*.^31^	Diogenes *et al*.^28^
Country	Argentina	The United States	Australia	Egypt	The Gambia	The Gambia	The Gambia	The Gambia	Brazil
Age	5–9 years	3 months and 2 years	4–7 years	6, 9, and 12 months	2, 13, and 52 weeks	5–10 years	8–12 years	5–10 years	5 weeks
Systolic BP (mmHg) *n* = Ca/placebo	*n* = 254/260, MD (95% CI) −1.4 (−3.3, 0.5)	**3 months**: *n* = 130/130, mean (SD) 111.4 (14.3) versus 113.6 (12.6), *P* > 0.05 **2 years**: *n* = 35/18, 95.4 (7.6) versus 100.2 (7.9), *P* < 0.05	*n* = 95/91, MD (95% CI) −0.1 (−2.4, 2.3)			*n = *179/171, MD (95% CI) −0.10 (−1.89, 1.68)			
Diastolic BP (mmHg) *n* = Ca/placebo	*n* = 254/260, MD (95% CI) −0.4 (−2.0, 1.2)		*n* = 95/91, MD (95% CI) 0.5 (−1.6, 2.6)			*n* = 179/171, MD (95% CI) 0.10 (−1.46, 1.67)			
High systolic BP (>95th percentile) *n* = Ca/placebo	*n* = 254/260, RR = 0.59; 95% CI: 0.39, 0.90					*n* = 179/171, OR = 1.1; 95% CI: 0.4, 3.1			
High diastolic BP (>95th percentile) *n* = Ca/placebo	*n* = 254/260, RR = 0.80; 95% CI: 0.49, 1.30								
Height/length (cm) *n* = Ca/placebo	*n* = 257/261, MD (95% CI) 0.17 (−1.04, 1.37)			**6 months**: *n* = 17/25, mean (SD) 64.7 (2.9) versus 65.1 (4.0), *P* = 0.74 **9 months**: *n* = 44/39, 68.2 (3.5) versus 66.4 (4.1), *P* = 0.04 **12 months**: *n* = 17/17, 70.3 (2.8) versus 70.1 (3.3), *P* = 0.86	**2 weeks**: *n* = 257/263, mean (SD) 50.5 (2.1) versus 50.8 (2.1), *P* = 0.08 **13 weeks**: *n* = 253/257, 59.6 (2.5) versus 59.8 (2.6), *P* = 0.4 **52 weeks**: *n* = 242/254, 71.3 (3.0) versus 71.5 (3.4), *P* = 0.5	*n* = 180/171, MD (95% CI)*^a^* −0.71 (−2.38, 0.95)	*n* = 223/224, MD (95% CI)*^a^* −0.57 (−1.81. 0.68) F: MD % (SE)*^b^* −1.0 (0.5), *P* = 0.04 M: 0.5 (0.5), *P* = 0.3	*n* = 141/149, MD (95% CI)*^a^* −0.24 (−2.06, 1.57)	*n* = 30/26, adjusted mean (SD) 54.1(1.7) versus 54.1(1.7), *P* = 0.99
Weight (kg) *n* = Ca/placebo	*n* = 257/261, MD (95% CI) 0.29 (−0.61, 1.19)			**6 months**: *n* = 17/25, mean (SD) 7.1 (0.9) versus 7.0 (0.9), *P* = 0.67 **9 months**: *n* = 44/39, 8.0 (0.9) versus 7.7 (1.1), *P* = 0.22 **12 months**: *n* = 17/17, 8.2 (0.9) versus 8.8 (0.9), *P* = 0.06	**2 weeks**: *n* = 257/263, mean (SD) 3.3 (0.5) versus 3.3 (0.5), *P* = 0.6 **13 weeks**: *n* = 254/259, 5.8 (0.8) versus 5.8 (0.8), *P* = 0.7 **52 weeks**: *n* = 243/254, 7.9 (1.1) versus 7.9 (1.0), *P* = 0.7	*n* = 180/171 MD (95% CI)*^a^* −0.21 (−0.92, 0.50)	*n* = 223/224, MD (95% CI)*^a^* −0.20 (−0.90, 0.50) F: MD % (SE)*^b^* −3.3 (1.5), *P* = 0.03 M: 2.2 (1.6), *P* = 0.2	*n* = 141/149, MD (95% CI)*^a^* 0.00 (−0.79, 0.80)	*n* = 30/26, adjusted mean (SD) 4.4 (0.4) versus 4.1 (0.4), *P* = 0.29
MUAC (cm) *n* = Ca/placebo				**6 months**: *n* = 17/25, mean (SD) 13.6 (1.0) versus 12.6 (1.0), *P* = 0.007 **9 months**: *n* = 44/39, 113.6 (1.2) versus 12.9 (2.2), *P* = 0.07 **12 months**: *n* = 17/17 13.7 (0.9) versus 13.8 (1.2), *P* = 0.76	**2 weeks**: *n* = 257/263, mean (SD) 10.2 (0.9) versus 10.1 (0.9), *P* = 0.6 **13 weeks**: *n* = 254/259, 12.9 (1.1) versus 12.8 (1.2), *P* = 0.7 **52 weeks**: *n* = 242/253, 13.4 (1.3) versus 13.3 (1.2), *P* = 0.1		*n* = 223/224, MD (95% CI)*^a^* −0.06 (−0.33, 0.21) F: MD % (SE)*^b^* −1.8 (1.0), *P* = 0.04 M: 2.0 (1.0), *P* = 0.05	*n* = 141/149, MD (95% CI)*^a^* 0.66 (−0.29, 0.42)	
TST (mm) *n* = Ca/placebo					**2 weeks**: *n* = 257/263, mean (SD) 57 (13) versus 57 (12), *P* = 0.9 **13 weeks**: *n* = 251/258, 77 (16) versus 76 (16), *P* = 0.7 **52 weeks**: *n* = 243/252, 72 (15) versus 70 (16), *P* = 0.1		*n* = 223/224 MD (95% CI)*^a^* 0.30 (−3.18, 3.78)	*n* = 141/149, MD (95% CI)*^a^* 0.07 (−0.35, 0.49)	
Lean body mass							*n* = 223/224, MD (95% CI)*^a^* −0.14 (−0.67, 0.39) F: MD % (SE)*^b^* −2.4 (1.4), *P* = 0.02 M: 1.1 (1.4), *P* = 0.4		
HC (cm)				**6 months**: *n* = 17/25, mean (SD) 41.7 (1.7) versus 41.7 (1.4), *P* = 0.94 **9 months**: *n* = 44/39, 42.2 (1.2) versus 42.0 (1.5), *P* = 0.50 **12 months**: 17/17, 42.8 (1.7) versus 42.3 (1.5), *P* = 0.42	**2 weeks**: *n* = 256/262, mean (SD) 35.4 (1.4) versus 35.5 (1.4), *P* = 0.8 **13 weeks**: *n* = 249/257, 39.9 (1.5) versus 40.0 (1.3), *P* = 0.9 **52 weeks**: *n* = 242/254, 44.3 (1.5) versus 44.4 (1.4), *P* = 0.7				

^*a*^ Original results presented by gender combined in RevMan.

^*b*^ Linear model, including sex, supplement, current age, and length at 52 weeks and sex–supplement interaction.

BMI, body mass index; BP, blood pressure; F, female; HC, head circumference; M, male; MD, mean difference; MUAC, mid‐upper arm circumference; TST, triceps skinfold thickness.

The Australian study^26^ reported data on offspring BP at 4–7 years of age and the Gambian follow‐up study^29^ at 5–10 years of age (Table 1). Neither study observed significant associations between calcium supplementation during pregnancy and offspring BP (Table 2). In the study from the United States, there was no statistically significant difference in systolic BP at 3 months of age between offspring of calcium and placebo group mothers, but at 2 years of age, the children of supplemented mothers presented with 4.8 mmHg lower mean systolic BP (*P* < 0.05).^25^ No statistically significant difference was found between the groups in diastolic BP at 2 years (Table 2). The latter study was assessed as having high risk of attrition bias.

The Argentinean study on a one‐site subpopulation of a four‐center trial reported no statistically significant difference between the calcium and placebo groups in the mean systolic or diastolic BP or the proportion of children with high (≥95th percentile) diastolic BP at 5–9 years.^24^ However, the proportion of children with high systolic BP was lower in the calcium group than in the placebo group (relative risk (RR) = 0.59; 95% CI: 0.39–0.90). Statistically significant effect of calcium on systolic BP was observed especially among children with body mass index (BMI) in the two upper quartiles of the study population. The mean differences (95% CI) in systolic BP between calcium and placebo groups were −3.2 (−6.3 to −0.1) mmHg in the third quartile and −5.8 (−9.8 to −1.7) mmHg in the highest quartile, and the RRs (95% CI) for high systolic BP were 0.37 (0.12–1.10) and 0.43 (0.26–0.71), respectively. No significant effect of maternal calcium supplementation during pregnancy on systolic BP was observed in the two lower BMI quartiles.

**Figure 4:**
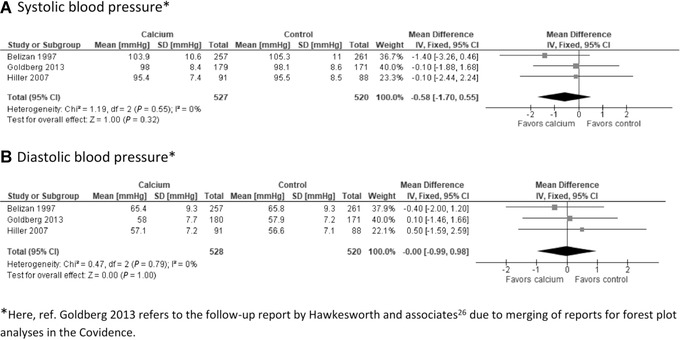
The effect of calcium supplementation on offspring blood pressure at 4–10 years of age.

A meta‐analysis combining results from three trials showed no significant effect of calcium supplementation on offspring mean systolic or diastolic BP at 4–10 years of age (Fig. 4). No forest plot analysis was undertaken for high systolic BP because the Gambian study reported this outcome at 5–10 years of age in 4% of the children but gave no exact numbers of children with a high systolic BP in the calcium and placebo groups.^29^ The effect of the intervention on offspring high systolic BP was interpreted as a possible positive effect on the basis of the finding in one moderate‐ to high‐quality study.^24^

##### Height and weight

Data on offspring height (or length) and weight were presented in seven reports from Argentina,^24^ Egypt,^27^ the Gambia,^23^, ^29^, ^31^ and Brazil,^28^ comprising 287 children in the calcium and 289 in the placebo group at 2–5 weeks, 287 and 293 children at 9–12 months, and 480 and 485 children at 5–12 years, respectively (Tables 1 and 2). Three reports were assessed as having a high risk and three an unknown risk of attrition bias. Other risk of bias was considered high in four and as unknown in two of these reports (Figs. 2 and 3).

Most studies reported no significant effect of maternal calcium with or without vitamin D on height or weight (Table 2). In one study, the mean length of the infants at 9 months of age was greater among offspring of supplemented mothers than those of the placebo group (mean (SD): 68.2 (3.5) versus 66.4 (4.1) cm, *P* = 0.04), but no difference in mean length was found at the ages of 6 or 12 months.^27^

The Gambian study using high‐dose calcium supplementation during pregnancy suggested some sex‐specific alterations in growth trajectories at age of 8–12 years.^30^ Female offspring of calcium‐supplemented mothers were shorter and lighter than those whose mothers received placebo (mean difference (SE) % in height = −1.0 (0.5) % and in weight = −3.3 (1.5) %), tested in linear models adjusted for potential confounders. Instead, accelerated growth was found among males. Consistent with this, the same research group found a higher mean plasma IGF1 concentration in boys and lower IGF1 concentration in girls of calcium‐supplemented mothers at 5–10 years of age.^31^ The sex–supplement interaction was statistically significant (*P* = 0.001).

**Figure 5:**
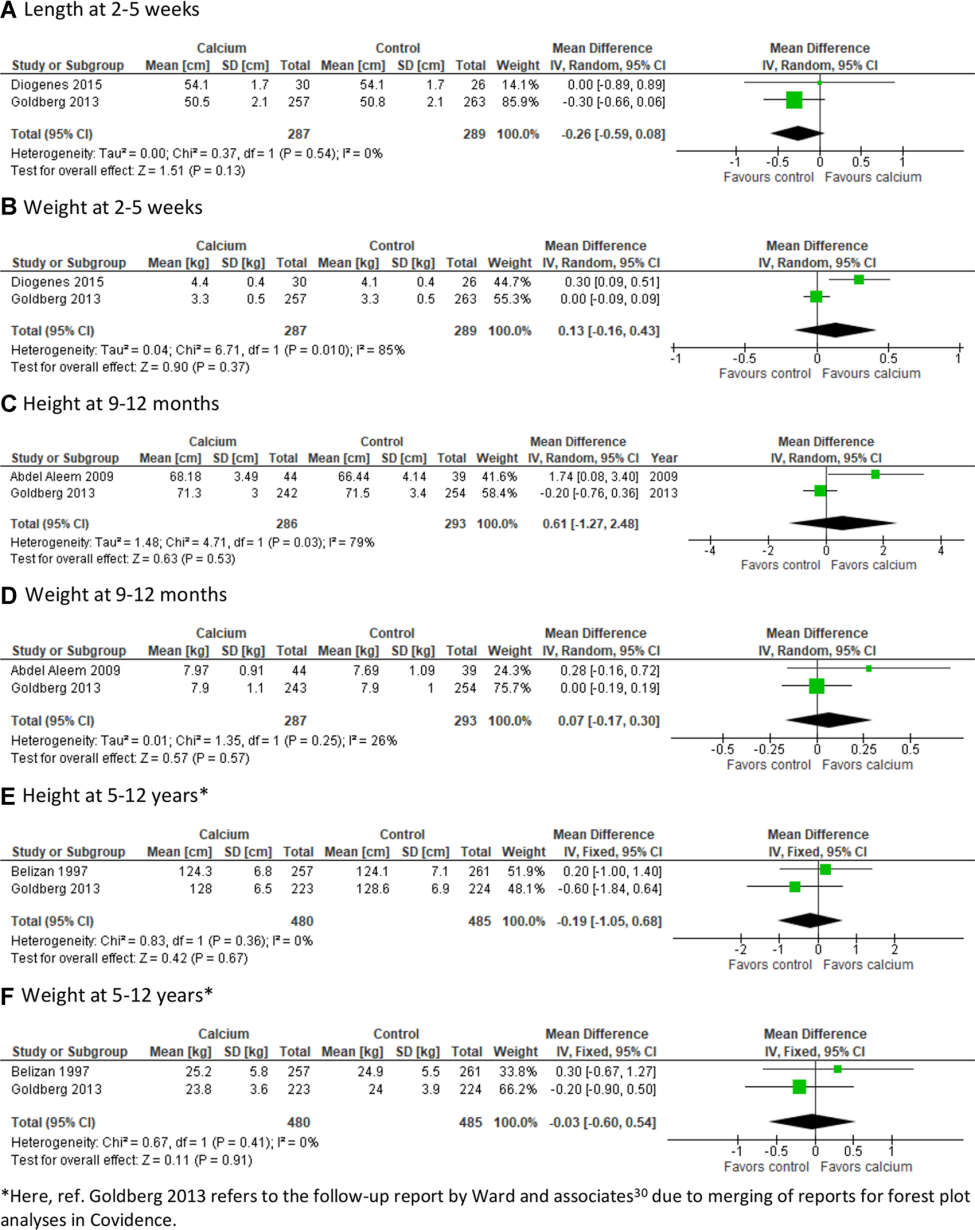
The effect of calcium supplementation on length/height and weight at 2–5 weeks, 9–12 months, and 5–12 years of age.

Meta‐analyses showed no significant effect of calcium supplementation on height (or length) or weight at 2–5 weeks of age, 9–12 months of age, or 4–12 years of age (Fig. 5). There was substantial heterogeneity between the studies especially in the younger age groups. The effect of the intervention on offspring height and weight was interpreted as unknown because of inconclusive published research.

#### Secondary outcomes

Four reports from Egypt^27^ and the Gambia^23^, ^30^, ^31^ presented data on MUAC, comprising 301 cases in the calcium and 302 in the placebo group by 1 year of age, and in partially overlapping populations of 141–223 and 149–224 cases at 5–12 years, respectively (Table 2). There were three Gambian follow‐up reports on triceps skinfold thickness^23^, ^30^, ^31^ at different ages, with 141–257 participants (54–99% of the total population) in the calcium group and 149–263 participants (56–99% of the total population) in the placebo group; one report on lean body mass (*n* (Ca/placebo) = 223/224) at 8–12 years;^30^ and two studies^23^, ^27^ (from Egypt and the Gambia) on head circumference (*n* (Ca/placebo) = 301/302) during the first year of life (Table 2).

No significant associations between maternal calcium supplementation and offspring triceps skinfold thickness, lean mass, or head circumference were observed in any of the studies. In the Egyptian study, MUAC was greater at 6 months in the calcium group than in the placebo group (mean (SD) = 13.6 (1.0) versus 12.6 (1.0), *P* = 0.007), but was similar at 9 and 12 months^27^ (Table 2).

Again, the Gambian follow‐up study suggested sex‐specific differences in MUAC at 8–12 years.^30^ Male offspring of mothers in the calcium group had greater MUAC (mean difference (SE) = + 2.0 (1.0) %, *P* = 0.05) and fat mass (+11.6 (5.1) %, *P* = 0.02) compared with the placebo group males, tested in linear models with sex, supplement, current age, length at 52 weeks, and sex–supplement interaction.

**Figure 6:**
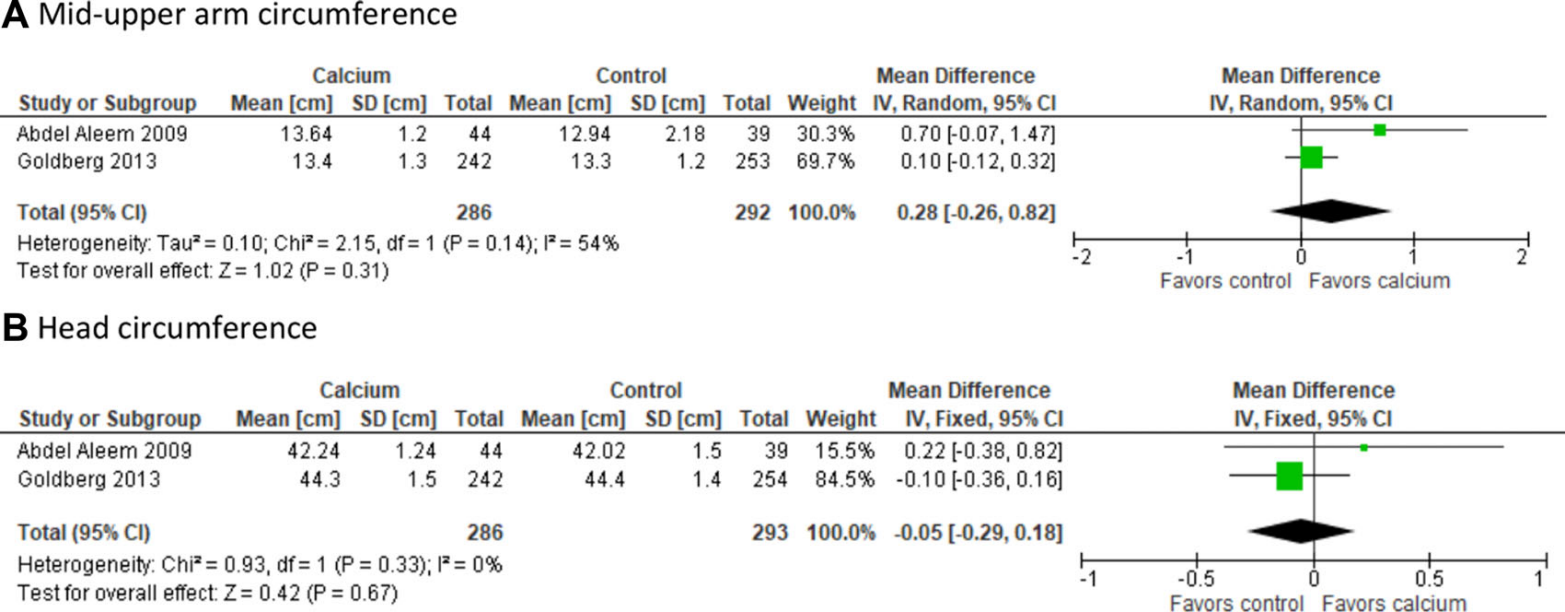
The effect of calcium supplementation on offspring mid‐upper arm and head circumference at 9–12 months of age.

Meta‐analysis showed no significant effect of calcium on MUAC or head circumference at 9–12 months of age (Fig. 6). Marked heterogeneity between studies was observed in the MUAC analysis. The effects of the intervention for offspring MUAC, triceps skinfold thickness, lean body mass, and head circumference were interpreted as unknown because of inconclusive published research.

## Discussion

### Summary of main results

On the basis of the results of one study,^24^ high‐dose calcium supplementation during pregnancy may decrease the risk of high systolic BP in childhood.

According to our systematic review on data from RCTs, calcium supplementation of mothers during pregnancy seemed not to show any significant benefit on length, weight, and head circumference growth of the offspring up to age of 8–12 years. A greater length and MUAC at 6 months of age in the offspring of the calcium‐supplemented mothers was a finding of unknown significance in one single study.^27^ The effect of calcium on MUAC was otherwise not significant. One study group reported some sex‐specific differences in maternal supplement effects on IGF1 levels and growth trajectories in later childhood, that is, slower growth among females and accelerated growth among male offspring of calcium‐supplemented mothers.^30^, ^31^

Calcium supplementation was combined with vitamin D in only one study, including less than 50 mothers in the intervention and control groups and, thus, providing insufficient data for any conclusions or subgroup analysis.

No RCTs were available on glucose intolerance, plasma lipid profile, cerebral palsy, developmental delay, intellectual impairment, and behavioral/learning difficulties of the offspring.

### Quality of evidence

The study populations were originally derived from six large RCTs, where the primary objectives were to examine the effect of calcium supplementation during pregnancy on hypertensive disorders in pregnancy. Only one of them had offspring follow‐up as an objective, that is, later infant growth as a secondary outcome.^23^ All original trials were assessed as having a low risk of selection bias. However, the selection of subpopulations for the follow‐up studies led to some significant differences between participants and nonparticipants in some maternal background characteristics, and in one study in offspring birth characteristics between the calcium supplementation group and control group participants. High attrition bias was a significant problem, ranging even between 47% and 89% in four studies. Information on potential confounders, including dietary habits, calcium and vitamin D intake, or physical activity of the participating children, was lacking. Some reported maternal compliance, dietary calcium intake,^26^ and information on breastfeeding,^26^, ^27^ while others did not. These factors may have significant effects on the studied outcomes. The intervals between randomization and ages at follow‐up varied, which makes comparison between studies challenging. We tried to take this into account by performing analyses in different age groups.

### Potential biases in the review process

The searches were planned in close collaboration with an experienced information specialist who also performed the searches. In addition, we tried to account for the risk of excluding eligible studies from the review by using several widely used databases and clearly defined inclusion criteria for the studies. The screening researchers had expertise in perinatology and neonatology. The members of the review team were not involved in any of the studies. No post‐hoc analyses were needed.

### Agreements and disagreements with other studies and reviews

Two systematic reviews have suggested an association between maternal calcium intake during pregnancy and offspring BP.^37^, ^38^ Both reviews also included three observational studies^39^, ^40^, ^41^ and acknowledged the challenges in the sample sizes and methodological problems. Our review focused on published results of RCTs. Follow‐up of the Project Viva cohort (eastern Massachusetts, 1999–2002 recruitment) has suggested an association between higher second‐trimester supplemental maternal calcium intake and lower systolic BP in the offspring at 6 months old,^40^ but not at 3 years old.^42^

Our observation on the possible protective effect of maternal calcium supplementation against high offspring BP was based on the Argentinean study,^24^ where the effect of calcium on BP seemed to increase with increasing offspring BMI. The evidence was interpreted as a possible positive effect. Another study^26^ (from Australia) did not find any association between calcium supplementation and offspring BP at 4–7 years old, and neither did the follow‐up of a Gambian study.^29^ In the latter, the effect of maternal intervention seemed not to be modified by offspring BMI. The authors suggested that the children recruited into the follow‐up may have been too young to show marked differences in cardiovascular risk factors, or the timing of intervention might have been too late in relation to organ development. The Gambian study children were also relatively lean, with a low prevalence of metabolic syndrome risk factors.^29^ The interpretation of the smaller Australian study^26^ and another one from the United States^25^ was hampered by a high risk of attrition bias.

Some authors have suggested that the effect of supplemental calcium on offspring BP might be independent of birth weight, possibly through intrauterine programming of calcium‐regulating hormones.^24^, ^25^ In a prospective Danish cohort study on 2434 women and 2217 children, the offspring BP trajectory until 5 years old was associated with maternal BP in pregnancy, independent of maternal and offspring covariates.^42^ Concentrations of placental growth factor at 28 weeks of gestation have correlated inversely to maternal gestational BP trajectory, independent of the diagnosis of pregnancy‐induced hypertension, and have been suggested to be a mediator of cardiovascular health in pregnancy.^43^

The result of our systematic review on the effect of calcium supplementation during pregnancy on the growth of offspring during infancy and later childhood showed mostly no effect on growth parameters. One research group has, however, reported sex‐specific differences in plasma IGF1 concentration and growth in later childhood.^30^, ^31^ Female and male offspring might differ in their susceptibility to changes in maternal diet. These data need to be confirmed. According to our review, the evidence for the effect of maternal calcium supplementation during pregnancy on the growth of offspring has to be regarded as unknown because of inconclusive published research.

### Conclusions

#### Implications for practice

Calcium supplementation during pregnancy may reduce the risk of high systolic BP in the offspring, especially in those with high BMI, but this finding needs more support from other follow‐up studies. Although the prevalence of elevated BP is quite low in children, it may anticipate later cardiovascular and metabolic problems in adolescence and adulthood. If the negative association between maternal calcium supplementation and high BP in the offspring were confirmed, this might suggest a benefit for public health.

The effect of maternal high‐dose calcium supplementation during pregnancy on the later growth parameters of the offspring seems to remain unknown owing to conflicting or insufficient data, and thus no implications for practice cannot be given.

While the introduction of routine prenatal calcium supplementation may be warranted in some settings due to its effect on maternal hypertensive diseases (and possible preterm delivery), its implementation would not be motivated by any known effects on later childhood outcomes.

#### Implications for research

The research on the effects and mechanisms of calcium exposure during fetal period on the offspring BP needs to be strengthened by further studies.

Further studies on growth need to be designed also for detecting possible sex‐specific differences in growth and body composition, because some differences in later childhood have been suspected. If the different susceptibility to maternal diet during pregnancy between girls and boys is confirmed, the mechanisms behind this need to be explored.

Limited available data from RCTs do not provide sufficient evidence to conclude that prenatal calcium supplementation influences offspring health outcomes beyond the newborn period. Lack of available research data on the effects of calcium supplementation during pregnancy on long‐term metabolic and neurodevelopmental outcome of the offspring are, however, of concern. Potential adverse effects of the intervention on these outcomes need to be investigated further, especially in settings, such as South Asia and Africa, where prenatal calcium supplementation is introduced on the basis of practice guidelines or as part of large‐scale public health programs.

## Author contributions

P.K. participated in designing and writing the study protocol, screened the studies, and had primary responsibility in analyzing the data and writing the manuscript. K.T. participated in designing and writing the study protocol, screened the studies, and participated in analyzing the data and writing the manuscript. J.I. performed the literature searches and participated in writing the manuscript. R.O. participated in designing and writing the study protocol and revising the manuscript. U.A. participated in designing and writing the study protocol, interpreting the analyzed data, and writing the manuscript. P.A. participated in designing and writing the study protocol, interpreting the analyzed data, and writing the manuscript. O.T. participated in designing and writing the study protocol, interpreting the analyzed data, and writing the manuscript.

## Competing interests

The authors declare no competing interests.

### Peer review

The peer review history for this article is available at https://publons.com/publon/10.1111/nyas.14729.

## Supporting information


**Appendix S1**. Full search strategies.

**Appendix S2**. Coding for the interpretation of the available evidence.
