# Supplementary material for: Calcium supplementation during pregnancy and long‐term offspring outcome: a systematic literature review and meta‐analysis
Source: Ann N Y Acad Sci. 2022 Jan 3;1510(1):36–51. doi: 10.1111/nyas.14729 (PMC9304138; doi:10.1111/nyas.14729)
Supplement: Supplementary file 2 — Appendix S2. Coding for the interpretation of the available evidence. [file NYAS-1510-36-s002.docx]

| Standardized statement | Situations included |
| --- | --- |
| Unknown effect: Insufficient published research on the intervention’s effect on the outcome. | No RCTs, one low quality RCT with any result, or |
|  | One moderate-to-high quality RCT where 95% CI of the RR includes 1, or |
|  | Only narrative reporting |
| Unknown effect: Inconclusive published research on the intervention’s effect on the outcome. | At least two RCTs, 95% CI of the point estimate for a relative risk crosses widely on both sides of 1 (ranges from <0.5 to >2) |
| Positive effect: The intervention likely reduces the risk of the adverse outcome. | At least two moderate-to-high quality RCTs included in a meta-analysis or IPD analysis, 95% CI of the point estimate of the RR is entirely below 1 |
| Possible positive effect: The intervention may reduce the risk of the adverse outcome. | At least two RCTs included in a meta-analysis or IPD analysis, 95% CI of the point estimate of the RR is entirely below 1, but there is concern about the quality of the data, or |
|  | at least two moderate-to-high quality RCTs included in a meta-analysis or individual patient data analysis, 95% CI of the point estimate of the RR includes 1 but 90% CI of the point estimate of the RR is entirely below 1, or |
|  | One moderate-to-high quality RCT, 95% CI of the point estimate of the RR is entirely below 1 |
| No positive effect: The intervention is not likely to reduce the risk of the adverse outcome. | Other situations, including meta-analysis results suggestive of harm |

Appendix S2. Coding for the interpretation of the available evidence (modified from Ref. 22).
